# Supplementary figures and images for: E-cigarette aerosol exposure of pulmonary surfactant impairs its surface tension reducing function
Source: PLoS One. 2022 Nov 9;17(11):e0272475. doi: 10.1371/journal.pone.0272475 (PMC9645651; doi:10.1371/journal.pone.0272475)

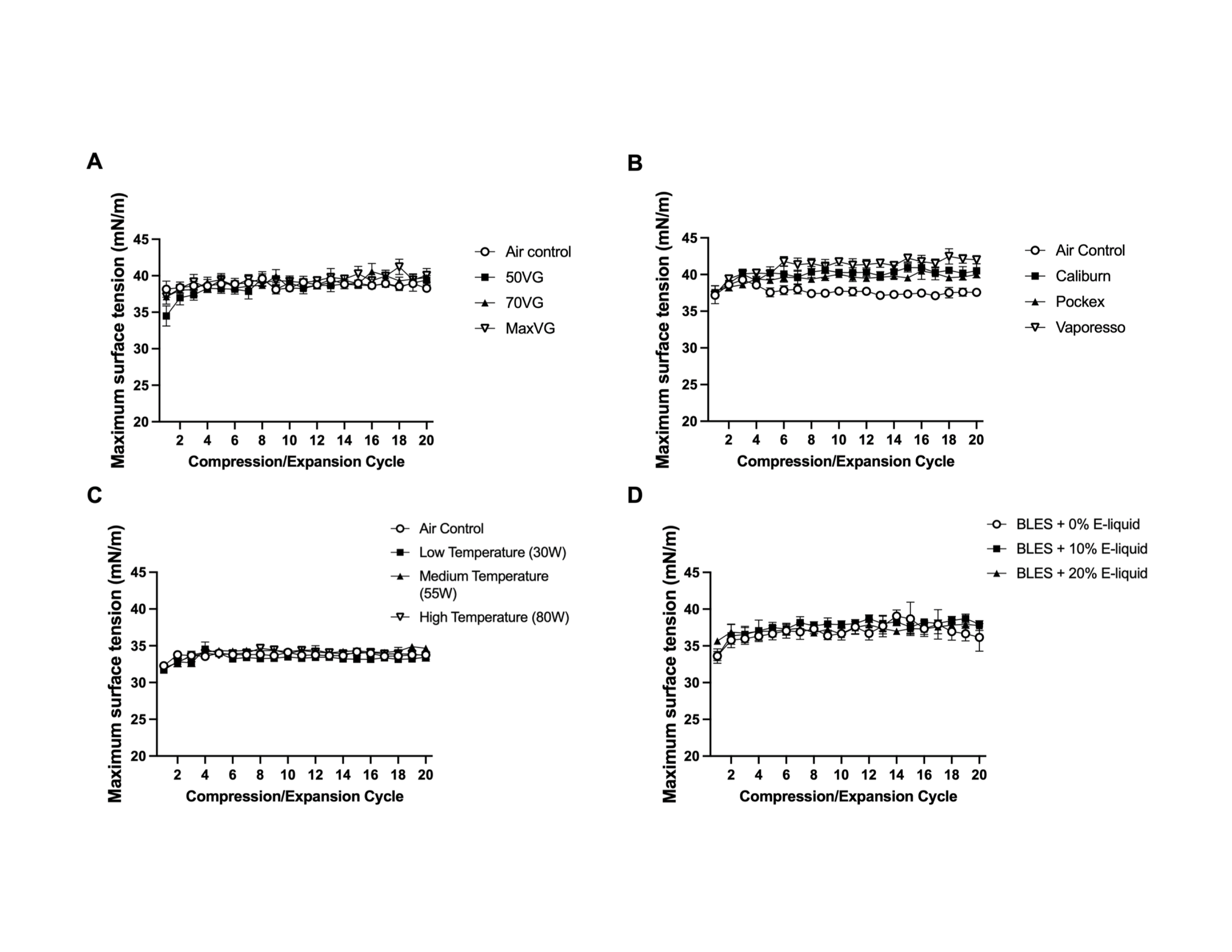

Supplement: S1 Fig — A-D. Maximum surface tensions of experimental variables. Maximum surface tensions (± SEM) across 20 compression/expansion cycles for BLES exposed to three experimental variables, compared to air control. A. Three different vehicle compositions with increasing VG content. B. Three different EC devices. C. Three different wattages set on a programmable EC to alter aerosol temperature. D. BLES mixed with three different volumes of vehicle e-liquid. N = 3 *P<0.05 compared to air control. (TIF) [file pone.0272475.s001.tif]

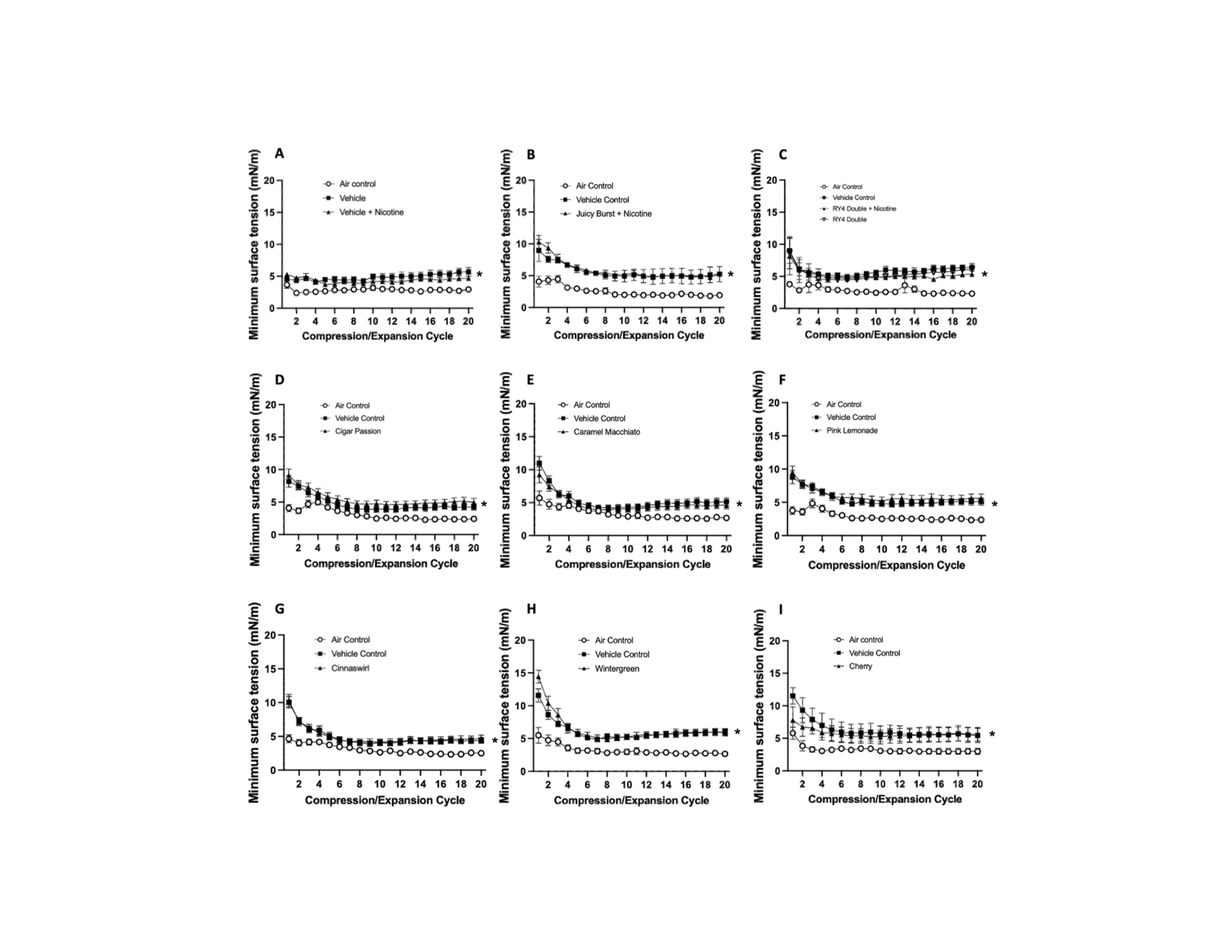

Supplement: S2 Fig — A-I. Minimum surface tensions (± SEM) across 20 compression/expansion cycles for BLES exposed to nine flavourings/additives compared to unflavoured vehicle control. N = 3. (TIF) [file pone.0272475.s002.tif]

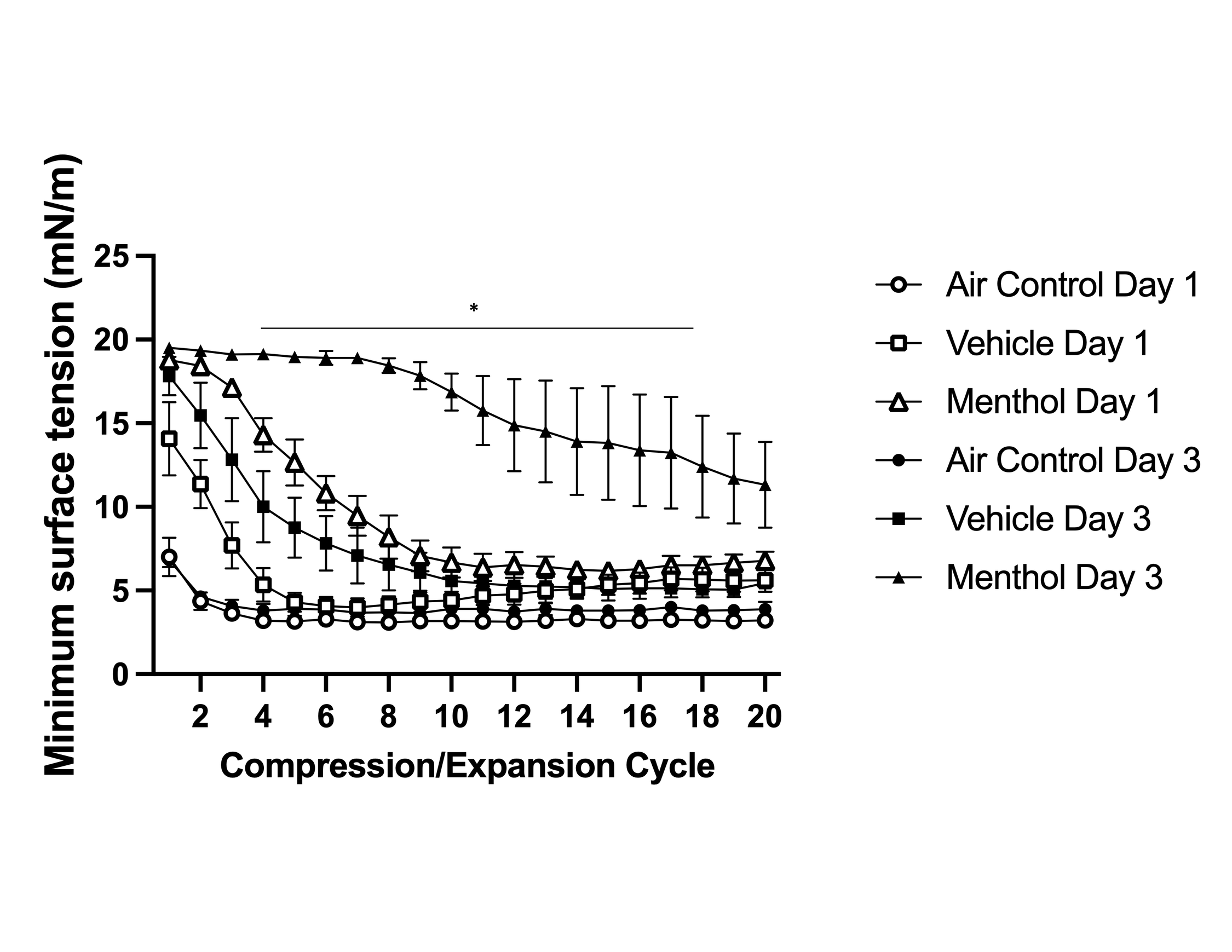

Supplement: S3 Fig — N = 3 *P<0.05 compared to day one. (TIF) [file pone.0272475.s003.tif]

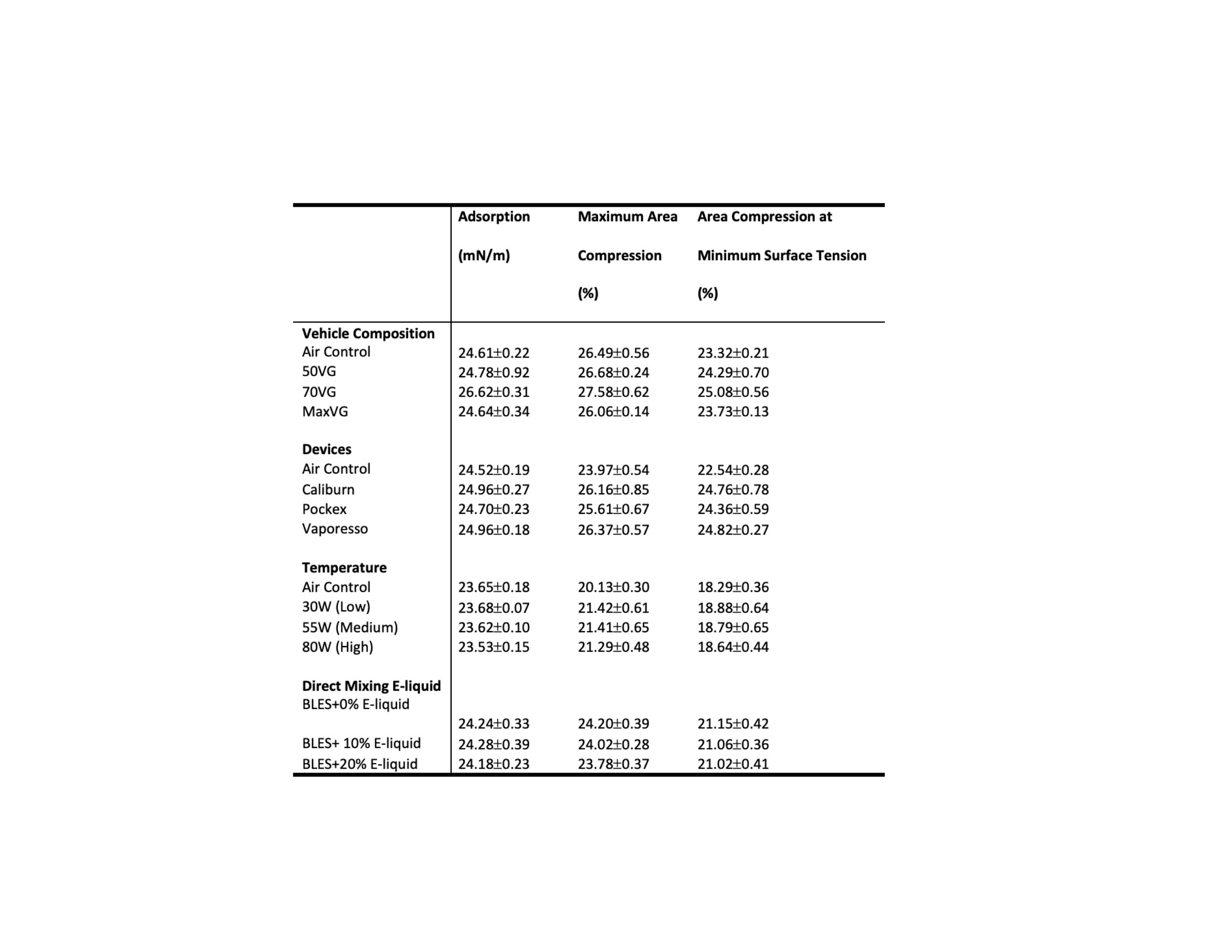

Supplement: S1 Table — Average adsorption and percent compression (±SEM) for control and exposed groups under four experimental conditions: changing vehicle composition, using different EC devices, varying temperature through increasing wattage, and mixing of non-aerosolized e-liquid directly with BLES. (TIF) [file pone.0272475.s004.tif]
